# Assessment of patient knowledge and perceptions towards orthodontic treatment in the Aljouf Region, Saudi Arabia: a cross-sectional study

**DOI:** 10.7717/peerj.18516

**Published:** 2024-11-13

**Authors:** Ayidh Muflih Alqahtani, Ashokkumar Thirunavukkarasu

**Affiliations:** Department of Family and Community Medicine, College of Medicine, Jouf University, Sakaka, Aljouf, Saudi Arabia

**Keywords:** Knowledge, Perception, Orthodontic treatment, Malocclusion

## Abstract

**Background:**

In orthodontic treatment, it is most important to assess the patient’s perspective of malocclusion because these perceptions are fundamental to determining the patient’s demands and level of treatment satisfaction. This study was done to assess the knowledge and perception of residents of the Aljouf Region of Saudi Arabia seeking orthodontic treatment. Furthermore, we determined the factors associated with the patients’ knowledge and perception of orthodontic treatment.

**Methods:**

This cross-sectional study was conducted using a pre-tested and validated questionnaire among patients seeking orthodontic treatment who were registered at King Abdulaziz Hospital, Sakaka, and primary health centers. We identified the associated factors using binomial logistic regression analysis. Finally, we applied Spearman’s correlation test to identify the correlation between knowledge and perception.

**Results:**

A total of 282 individuals participated in the current study, of which 53.3% were female and 46.1% were male. About one-third (36.9%) of patients had a low level of knowledge, 35.5% had a medium level of knowledge, and 27.7% had a high level of knowledge regarding orthodontic treatment. A total of 20.6% had low perceptions, 42.9% had medium perceptions, and 36.5% had high perceptions of orthodontic care. A significant correlation was found between age and gender, knowledge (*p* = 0.001), and perception (*p* = 0.040). Moreover, we observed a positive correlation between knowledge and perception (Spearman’s rho = 0.297, *p* = 0.001).

**Conclusion:**

People in Aljouf have poor knowledge and medium perception regarding orthodontic treatment. We recommend that the concerned authorities make a health educational plan for the patients seeking orthodontic treatment.

## Introduction

Malocclusion in dentistry refers to any aberration in the proportions or location of teeth that is not consistent with the other dental arches. Malocclusion can be considered an oral health condition that increases the risk of dental caries, periodontal diseases, psychological issues, potential temporomandibular disorders, and other serious conditions that impact oral hygiene and well-being (FDI World Dental Federation, 2019; Gaikwad et al., 2014; Rai et al., 2021). Malocclusion, in contrast to other dental issues, is a range of varying degrees of occlusion abnormalities rather than a disease. However, the most severe malocclusions can be severely debilitating in terms of how they affect daily life, and in many countries, public oral health care programs cover their treatment (Ghodasra & Brizuela, 2024). The degree of malocclusion can be measured to determine how much the case deviates from normal occlusion. It has been found that the severity of malocclusion is correlated with the requirement for orthodontic treatment (Zasčiurinskienė et al., 2023b).

Globally, malocclusion is a prevalent phenomenon. A systematic review published in 2020 indicated that there was a 56% global prevalence of malocclusion, regardless of gender. Africa (81%) and Europe (72%) had the highest prevalence, followed by America (53%) and Asia (48%) (Lombardo et al., 2020). While assessing the burden of malocclusion in Saudi Arabia, the findings of a recent systematic review published in 2021 demonstrated that malocclusion affected 72% of the candidates. The corresponding prevalence rates for Class I, Class II, and Class III malocclusion were 66.51%, 17.70%, and 15.79%, respectively. Class I malocclusion was most common in both male and female participants followed by Class II and III malocclusion (Devanna et al., 2021). This signifies the burden of malocclusion in the Saudi population and their need to seek orthodontic treatment.

Although orthodontic treatment is the preferred course of action for treating various malocclusions, the decision to pursue orthodontic treatment is a complex one (Alharbi & Taju, 2024). Both objective and subjective factors are taken into consideration when selecting patients for orthodontic treatment. Subjective orthodontic treatment need is influenced by various factors, including the patient’s self-perception of necessitating orthodontic treatment, while objective orthodontic treatment need is established by the clinical findings of specialists. Studies have demonstrated that aesthetic features such as teeth alignment, spacing, and unattractive smiles are the most common reasons for seeking treatment, including re-treatment (Samsonyanová & Broukal, 2014; Tashkandi et al., 2023). More in-depth research has revealed that protrusion and spacing rank first and second amongst complaints between adults in their 20s and 30s, respectively. Nonetheless, there is a difference between these two age groups: crowding was the third most prevalent complaint for people in their 30s, while an unattractive smile was the most prevalent complaint for people in the 20–29 age range (Alshammari et al., 2022). The number of patients seeking orthodontic treatment has grown over the past few decades, and with it have come higher demands for shorter treatment time frames and more aesthetically pleasing results. Various methods, including non-surgical and surgical ones, are employed as adjuncts to orthodontic treatment. The severity of the condition, necessity for premolar extractions, physician’s experience, and, of course, patient participation are some factors that may influence the duration of treatment (Apalimova et al., 2020).

Successful orthodontic treatment requires understanding patient perspectives of malocclusion, as these influence treatment satisfaction and outcomes (Mani et al., 2021). Research has demonstrated that individuals who possess greater knowledge about orthodontics tend to have more positive attitudes towards orthodontic treatment, which in turn increases the possibility of better clinical outcomes (Mathew et al., 2023).

Studies from Europe (Zasčiurinskienė et al., 2023a), India (Sri et al., 2022), and Egypt (El Mofty, 2022) reveal varied levels of awareness, attitudes, and knowledge of orthodontic treatment. While patients generally understand the aesthetic benefits, awareness of specific risks such as enamel demineralization and temporomandibular disorders remains low. Findings of a cross-sectional survey from Saudi Arabia’s Riyadh region demonstrated that the majority of research participants were well-informed regarding malocclusion and dental health, although women were more inclined than men to place importance on aesthetics (Gowdar et al., 2022). Furthermore, another cross-sectional study from Madinah, Saudi Arabia suggested that regarding malocclusion, the majority of participants demonstrated a high degree of awareness and understanding of issues unique to oral health. The perception of social and orthodontic treatment characteristics as obstacles to the acceptance of orthodontic care persisted even though females were considerably more knowledgeable and aware of malocclusion than males (Ashky et al., 2019). Because of the high burden of malocclusion and increased demand for orthodontic treatment among the Saudi population, this cross-sectional study was conducted with the following aims and hypothesis.

Aim:

The primary aim of this study is to assess the knowledge and perceptions of patients seeking orthodontic treatment in the Aljouf region, Saudi Arabia. Furthermore, we aimed to determine the factors associated with the patients’ knowledge and perception of orthodontic treatment.

Hypothesis:

We hypothesize that patients seeking orthodontic treatment in the Aljouf region have limited knowledge and varying perceptions of orthodontic treatment. Furthermore, demographic factors such as age, gender, and educational background are expected to influence these levels of knowledge and perception.

## Materials and methods

### Study description

This oral health-related survey used a cross-sectional design that was conducted in the Al-jouf region of Saudi Arabia from March 2024 to June 2024. Al-jouf consists of four governates, namely Skaka, Dwamat Al-Jindal, Tabarjil, and Al-Qurrayat. The research team conducted this study only in the Skaka and Dwamt Al-jindal governates. According to the statistics authority of Saudi Arabia, this region has a population of about half a million people. In this region, facilities for orthodontic treatment are available at King Abdulaziz Specialty hospital (KAASH). Furthermore, the primary health centers refer patients to this specialty hospital for orthodontic care. We included male and female patients aged 18 years and above seeking orthodontic treatment. These patients were either registered at KAASH or referred to KAASH from the primary health centers. We excluded non-Saudi nationals, unwilling patients and those who did not belong to the Aljouf region. Verifying the name and age excluded duplicate/double registration. However, after exclusion, the collected data from the study participants were anonymous.

### Sample size and method

**Figure 1:**
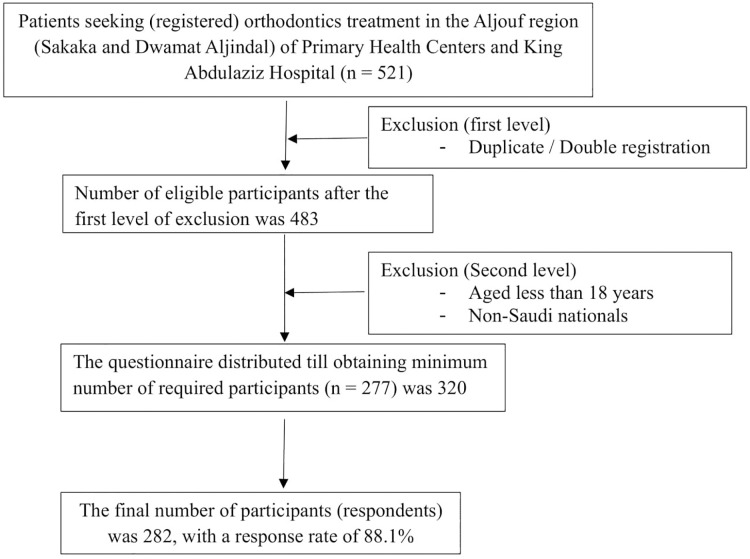
Sampling process flowchart.

We calculated the sample size using the Raosoft online sample size calculator. In this, we used 23.3% as expected proportion (p), 95% confidence interval (CI), 5% margin of error, and 80% of power. Since there have been few studies with varying degrees of results, we calculated the sample size based on the expected proportion obtained in the pilot study (23.3%). Applying these values, we got the minimum required sample size of 277. We distributed the questionnaire to 320 patients using a convenience sampling method to select the required participants for data collection. Overall, 282 patients responded (response rate = 88.1%). This method was chosen primarily due to the accessibility of patients who were actively seeking orthodontic treatment during the study period. The method allowed us to efficiently gather data from a population that directly aligned with the study’s objectives—patients seeking treatment. The sampling process of the present study is depicted in a flow chart (Fig. 1).

### Data collection procedure

We started the data collection process after obtaining ethical clearance from the Institutional Ethical Review Board of the Central Institutional Review Board, Ministry of Health, Saudi Arabia (approval no: 24-31 E, dated 17.03.2024) and other necessary approvals from KAASH and primary health center management. With the approval of the concerned authorities, the patients were communicated with and requested to fill out the Google form in the data collector’s personal device after obtaining written informed consent. All of the details related to informed consent were mentioned on the first section of the Google form. The questionnaire was directed to the patients who had undergone an initial consultation and registration at KAASH or primary health centers and were interested in orthodontics. This was done to factor in those respondents who had been provided with basic information about orthodontic treatment options but had not begun active treatment. This timing was chosen in order to evaluate patients’ knowledge and perceptions prior to the commencement of their treatment to make well-informed decisions. It is worth mentioning that most of the primary health centers in this region consist of a general dentistry department that treats common dental issues that can be treated as out-patient and patients are referred to higher-level centers if needed. Only participants who were aged 18 years and above and agreed to participate were allowed to go to the next pages of the Google form. If participants answered “yes” to the informed consent question, they were automatically directed to the next section of the questionnaire. However, if participants answered “no,” they were not allowed to proceed further, and the form did not grant them access to the remaining sections. The remaining pages of the form consisted of a structured questionnaire to collect data on participants’ background characteristics, knowledge related to orthodontic treatment, and perceptions of orthodontic care. This data collection tool was prepared by the research team. The content validation was done by experts and investigators through a focused group discussion after reviewing the open sources pieces of literature (El Mofty, 2022; Ghodasra & Brizuela, 2024; Mathew et al., 2023). To achieve content validity, the questionnaire was presented to a panel of orthodontists, public health specialists, and survey designers. These experts gave feedback on each item of the questionnaires regarding their intended clarity, relevance, and comprehensiveness. The language, order, and inclusion of specific items were adjusted based on this feedback so that the tool would obtain the necessary and intended data. Originally, we developed this tool in English, and we followed the recommended methods for translation (Arabic) and back translation (English) to check whether the intended meaning was retained during the translation (McIlvenny et al., 1999; Tsang, Royse & Terkawi, 2017; Walde & Völlm, 2023). The developed Arabic tool was tested across 30 patients who were seeking orthodontic treatment and fulfilled all the eligibility criteria. This was done to check for face validity and reliability. At the end of the pilot test, all participants indicated that the questions were easy to understand and clear. Hence, we used the developed Arabic questionnaire for the main study. The average duration to complete the survey was seven minutes. The Cronbach’s alpha value obtained through the pilot study was 0.77 for knowledge and 0.83 for the perceptions section. The data collection tool consisted of three parts. The first part consisted of background characteristics of the patients. The second section was about patients’ knowledge towards orthodontic treatment, including indication and side effects (10 questions). In the knowledge section, the participants had to choose the best answer according to their knowledge. The correct answers were given 1 point, and the false answers were given 0 points. In the perception section (third part), the patients responded on a Likert scale ranging from strongly disagree (one point) to strongly agree (five points) to the 10 items. The total score of the knowledge and perception answers were calculated for further analysis. We divided the knowledge and perception scores into low (below 60% of total), medium (60% to 79% of total), and high (80% and more of total) (ALruwaili et al., 2022; Alzahrani et al., 2022).

### Data analysis

We utilized the Statistical Package for Social Sciences version 24 (V.24) for data exporting, coding, and analysis. The descriptive data related to patients’ background characteristics, knowledge, and perceptions towards orthodontic treatment was presented as a frequency and percentage. The quantitative data was presented as mean and standard deviation. Since the data did not meet the normality assumption (Shapiro–Wilk test), we applied Spearman’s rank correlation test. Finally, we applied binomial logistic regression tests (enter technique) to finalize the predictors for low/medium and high scores in knowledge and perception categories. A *p*-value less than 0.05 was considered a statistically significant value.

## Results

Of the 282 individuals who participated in the current study, the majority (53.3%) were female, between the ages of 18 and 30 (45.4%), private employees (42.6%), and had completed university or graduate studies (72%). Regarding income, the majority earned more than 7,000 Saudi Riyals (SAR) [1 USD = 3.75 SAR] per month (63.1%). Most of them were non-smokers (89.4%) and had not had previous orthodontic treatment done (67.4%) (Table 1).

**Table 1:** Demographic data of the participants (*n* = 282)

Variable	Number	Proportion
Age group		
18 to 30	128	(45.4)
31 to 40	99	(35.1)
41 and above	55	(19.5)
Gender		
Male	130	(46.1)
Female	152	(53.9)
Occupation:		
Government	90	(31.9)
Private	120	(42.6)
Unemployed	72	(25.5)
Education status:		
Up to high school	79	(28.0)
University/Graduate studies	203	(72.0)
Income		
Less than 5,000 SAR	58	(20.6)
5,000 to 7,000 SAR	46	(16.3)
More than 7,000 SAR	178	(63.1)
Smoking		
No	252	(89.4)
Yes	30	(10.6)
Previous orthodontic treatment		
No	190	(67.4)
Yes	92	(32.6)

Regarding the knowledge related to orthodontic treatment, the questions “Which type of orthodontic appliance is typically worn at night to prevent teeth from shifting?” (KQ2), “At what age is orthodontic treatment typically initiated?” (KQ5), and “What is one potential risk associated with orthodontic treatment?” (KQ8) were largely answered incorrectly (Table 2).

**Table 2:** Participants responses in knowledge category (*n* = 282).

Items	Correct answer		Wrong answer	
	*n*	%	*n*	%
What is the primary goal of orthodontic treatment? (KQ1)	232	82.3	50	17.7
Which type of orthodontic appliance is typically worn at night to prevent teeth from shifting? (KQ2)	105	37.2	177	62.8
What is a potential side effect of orthodontic treatment? (KQ3)	160	56.7	122	34.3
How often should you visit your orthodontist for adjustments during treatment? (KQ4)	189	67.0	93	33.0
At what age is orthodontic treatment typically initiated? (KQ5)	88	31.2	194	68.8
Which factor can influence the duration of orthodontic treatment? (KQ6)	191	67.7	91	32.3
What role do orthodontic retainers play after treatment? (KQ7)	223	79.1	59	20.9
What is one potential risk associated with orthodontic treatment? (KQ8)	127	45.0	155	55.0
What can be a consequence of not wearing orthodontic retainers as instructed? (KQ9)	217	77.0	56	23.0
How can orthodontic treatment impact overall oral health? (KQ10)	164	58.2	118	41.8

Regarding the perception of orthodontic care, the majority of the patients showed negative perception towards orthodontic care as the answers for eight questions out of 10 were ‘disagree or strongly disagree’. Only two questions (PQ3 and PQ4) had a ‘neutral’ response (Table 3).

**Table 3:** Participants responses in section (*n* = 282).

Item	Strongly agree *n* (%)	Agree *n* (%)	Neutral *n* (%)	Disagree *n* (%)	Strongly disagree *n* (%)
Orthodontic treatment aims to improve both the appearance and function of the teeth and jaws (PQ 1)	23 (8.2)	5 (1.8)	26 (9.2)	97 (34.4)	131 (46.5)
Orthodontic treatment effectively enhances smile aesthetics. (PQ 2)	19 (6.7)	5 (1.8)	38 (13.5)	99 (35.1)	121 (42.9)
The benefits of orthodontic treatment justify the associated costs in terms of overall value and long-term outcomes. (PQ 3)	20 (7.1)	24 (8.5)	101 (35.8)	78 (27.7)	59 (20.9)
Orthodontic treatment is accessible and affordable for most individuals. (PQ 4)	35 (12.4)	64 (22.7)	95 (33.7)	66 (23.4)	22 (7.8)
The discomfort experienced during orthodontic treatment is manageable and worthwhile. (PQ 5)	23 (8.2)	25 (8.9)	95 (33.7)	105 (37.2)	34 (12.1)
Orthodontic treatment is an essential investment in long-term oral health. (PQ 6)	16 (5.7)	18 (6.4)	54 (19.1)	101 (35.8)	93 (33.0)
Orthodontic treatment significantly boosts self-confidence. (PQ 7)	16 (5.7)	16 (5.7)	49 (17.4)	93 (33.0)	108 (38.3)
Orthodontic treatment significantly reduces the risk of future dental problems. (PQ 8)	20 (7.1)	12 (4.3)	76 (27.0)	97 (34.4)	77 (27.3)
Orthodontic treatment enhances the overall quality of life (PQ 9)	19 (6.7)	15 (5.3)	67 (23.8)	103 (36.5)	78 (27.7)
A final informed decision to undergo orthodontics treatment or not, will be taken by me after discussing with the dentist. (PQ 10)	11 (3.9)	19 (6.7)	49 (17.4)	98 (34.8)	105 (37.2)

After analyzing the correlation between knowledge and perception using Spearman’s Correlation coefficient, a significant difference between the two was found (*p* = 0.001) (Table 4).

**Table 4:** Spearman’s correlation analysis between knowledge and perception.

Variable	rho*/*p*-value
Knowledge – Perception	0.297 (0.001)

**Note:**

* Spearman’s correlation value.

When analyzing and categorizing data for knowledge, it was found that 36.9% of patients had low knowledge, 35.5% had medium knowledge, and 27.7% had high knowledge of orthodontic treatment. Regarding perception, 20.6% had low perception, 42.9% had medium perception, and 36.5% had high perception of orthodontic care. A significant association was found for the variables age with knowledge (*p* = 0.001) and perception (*p* = 0.040). Similarly, a significant correlation was found between the variable gender with knowledge (*p* = 0.008) and perception (*p* = 0.020) (Table 5).

**Table 5:** Binomial logistic regression analysis of knowledge and perceptions towards orthodontics treatment.

Variables	Total	Knowledge				Perception			
		Low/Medium (*n*)	High (*n*)	Adjusted OR (95% CI)*	*p*-value	Low/Medium (*n*)	High (*n*)	Adjusted OR (95% CI)*	*p*-value
Age group									
18 to 30	128	75	53	Ref		80	48	Ref	
31 to 40	99	81	18	1.524 [0.59–3.91]	0.381	58	41	1.757 [0.87–3.55]	0.117
41 and above	55	48	7	4.846 [2.04–7.54]	0.001	41	14	2.070 [1.16–4.28]	0.040
Gender									
Male	130	104	26	Ref		95	35	Ref	
Female	152	100	52	0.481 [0.28–0.83]	0.008	84	68	0.455 [0.28–0.75]	0.002
Occupation:									
Government	90	72	18	Ref		58	32	Ref	
Private	120	77	43	0.809 [0.38–1.71]	0.579	72	48	1.18 [0.61–2.27]	0.630
Unemployed	72	55	17	1.807 [0.93–3.49]	0.079	49	23	1.42 [0.77–2.63]	0.264
Education status:									
Up to high school	79	60	19	Ref		55	24	Ref	
University/Graduate studies	203	144	59	0.773 [0.43–1.41]	0.605	124	79	0.685 [0.39–1.19]	0.685
Income									
Less than 5,000 SAR	58	40	18	Ref		40	18	Ref	
5,000 to 7,000 SAR	46	35	11	1.185 [0.62–2.26]	0.607	28	18	0.746 [0.40–1.41]	0.364
More than 7,000 SAR	178	129	49	0.827 [0.39–1.76]	0.622	111	67	1.065 [0.55–2.07]	0.853
Smoking									
No	252	187	65	Ref		157	95	Ref	
Yes	30	17	13	0.455 [0.21–0.99]	0.046	22	8	1.664 [0.71–3.89]	0.239
Previous orthodontic treatment									
No	190	129	61	Ref		119	71	Ref	
Yes	92	55	37	0.732 [0.51–0.83]	0.009	60	32	1.119 [0.67–1.88]	0.672

**Note:**

* Adjusted with other variables of the study.

## Discussion

The current cross- sectional observational study was undertaken to assess the knowledge and perception for seeking orthodontic treatment among residents of Saudi Arabia’s Aljouf Region. The results of the current study reveal that the participants had low knowledge and negative perception towards orthodontic treatment.

There is a growing need for orthodontic treatment in the population of Saudi Arabia. A study in 2018 concluded among 670 (390 female and 280 male) secondary and high school students in Saudi Arabia, approximately one-quarter of the participants had severe or extreme need for orthodontic treatment (Alhummayani & Taibah, 2018). Previous studies have shown that the lack of orthodontic treatment in people of a particular geographic area may be due to several reasons such as literacy rate, lack of resources, and socioeconomic status (Awaisi, Asad & Mahmood, 2012; Feldmann et al., 2007; Zakirulla et al., 2019). In the current study, the majority of respondents received higher education and were in a higher bracket of earning. Thus, adequate information sources providing correct and reliable information regarding orthodontic treatment must be readily available to enable citizens to make informed decisions.

In this study, it is evident that the participants’ knowledge related to orthodontic treatment was ‘poor’, with the majority of the participants getting low scores. The question about the ‘potential risk associated with orthodontic treatment’ was answered incorrectly by 55% of participants.

Similarly, the question ‘what age is the treatment initiated?’ was incorrectly answered by the majority of the respondents (68.8%). According to the American Association of Orthodontics, children should receive their first orthodontic consultation at the age of 7 (Taibah & Al-Hummayani, 2017). In the case of children, the awareness of parents regarding the appropriate timing for orthodontic consultation influences the decision towards the treatment (Marques et al., 2009). Similarly, the question ‘what type of orthodontic appliance is typically worn in the night?’ (62.8%) was incorrectly answered by the majority of the participants. The results of the current study are somewhat similar to those of others who found moderate to high parental knowledge related to their children’s occlusal condition and need for orthodontic treatment (Kim, 2017). Another study showed that the knowledge of patients regarding the pain that may be experienced during orthodontic treatment was average (Amalia, Anggani & Ismaniati, 2011). Notably, in the current study, 56.7% respondents correctly answered the question on the potential side effects of orthodontic treatment.

Regarding the perception of orthodontic treatment, none of the responses indicated a positive perception. Only two responses (‘The benefits of orthodontic treatment justify the associated costs in terms of overall value and long-term outcomes’ and ‘Orthodontic treatment is accessible and affordable for most individuals’) had a ‘neutral’ response while all other items indicated a ‘medium’ perception as participants responded as either they ‘disagreed’ or ‘strongly disagreed’.

Among the demographic variables tested for the correlation with knowledge and perception, age (*p* = 0.001, *p* = 0.004) and gender (*p* = 0.008, *p* = 0.002) showed a significant association. In the current study, the majority of the participants were female (53.9%) within the age range of 18 to 30 years (45.4%). Thus, it can be said that younger females were more knowledgeable and had better perception towards orthodontic treatment than their counterparts. Younger female individuals, in general, have more knowledge regarding orthodontic treatment compared to older individuals. In a study by Kim (2017), it was found that 63.2% of younger Korean patients (in their 20s) had a positive interest in orthodontic treatment. Similar results were obtained by one study that found that the majority of female respondents in the Federal Territories of Kuala Lumpur and Putrajaya (64.1%) correctly answered questions in the knowledge domain about orthodontic treatment (Mathew et al., 2023). Another study conducted in Saudi Arabia showed a significant difference when comparing the mean scores of knowledge and attitude based on age (Althogbi et al., 2017).

In many countries, there is disparity in patient knowledge regarding orthodontic treatment. This is often influenced by cultural, socioeconomic, and educational factors. The results from the Aljouf region reflect the findings from studies in diverse regions such as Europe, Asia, and Africa, where patients tend to have a higher understanding of the aesthetic benefits but are often unaware of the full scope of risks and long-term implications of orthodontic treatment. The findings of a European cross-sectional study demonstrated that individuals’ awareness of periodontal disease is related to their interest in receiving orthodontic treatment (Zasčiurinskienė et al., 2023a). The results of an Indian cross-sectional study among 100 participants observed adequate awareness, attitude, and knowledge towards orthodontic treatment (Sri et al., 2022). Moreover, findings of an Egyptian survey revealed that the patients’ degree of knowledge of orthodontics as a specialty was higher than their level of knowledge regarding malocclusion; however, the awareness levels of males and females differed significantly (El Mofty, 2022). Amalia, Anggani & Ismaniati (2011) found that patients had average knowledge about pain side effects and good knowledge about soft tissue damage risks during orthodontic treatment. However, their understanding of potential enamel demineralization, tooth vitality loss, periodontal issues, root resorption, temporomandibular joint disorders, and relapse risks was low (Amalia, Anggani & Ismaniati, 2011). The findings from the present study and studies from other countries reflect a global need for continuous education for patients seeking orthodontic treatment, particularly in underserved regions where healthcare literacy is low. The variations across the countries also indicate that tailored public health initiatives, including those using digital platforms and social media for education, could be useful initiatives.

Overall, the results of the current study indicate poor knowledge and perception towards orthodontic treatment among people from Aljouf. These findings mirror similar results from various other regions in Saudi Arabia such as Jeddah (Alsaggaf et al., 2022), Hail (Alnaafa et al., 2020), and Al-Madinah in Saudi Arabia with moderate awareness among parents regarding orthodontic treatment (Almarhoumi et al., 2022). Similarly, parents in Dammam had poor knowledge about preventive orthodontics (Zakirulla et al., 2019). Given that social media is an effective tool to improve knowledge about oral hygiene and orthodontics, the development of knowledge-enhancing content and use of social media and smartphones is suggested (Siddiqui, Chia & Sharif, 2022). Further, studies must aim at analyzing the effectiveness of these measures to increase knowledge and improve perception towards orthodontic treatment in the Aljouf region.

### Limitations

We conducted this survey using standard methodology. However, some limitations need to be considered when interpreting the present study’s findings. First, we assessed the patients’ knowledge and perception in a single region. Hence, the present findings may not be useful to generalize throughout Saudi Arabia. Second, we used convenience sampling that might have introduced selection bias. This method limits the randomness of the sample and may affect the generalizability of the findings. Additionally, considering the nature of cross-sectional study design, it is difficult to establish causality or track changes in knowledge and perceptions over time. Finally, the reliance on self-reported questionnaires may introduce response bias, where participants might overestimate or underestimate their knowledge or perceptions.

## Conclusion

Based on the results of the current study, it can be concluded that individuals in Aljouf seeking orthodontic treatment possess limited knowledge and medium perceptions about the treatment. Both age and gender were associated with knowledge and perception of orthodontic treatment. Female and younger individuals seemed to have better knowledge and perception. Based on the findings of this study, the authors recommend that the targeted educational initiatives should be implemented to improve knowledge and perceptions regarding orthodontic treatment in the Aljouf region. Although patients receive basic information during the registration process, this information is insufficient to fully address their knowledge gaps regarding orthodontic care and the associated risks and benefits. Hence, it is essential to have continuous education, in addition to the initial information received, to make better-informed decisions. The use of social media and development of knowledge-enhancing content for the people of Aljouf, Saudi Arabia are suggested as it will help in reaching a broader audience and addressing common misconceptions.

## Supplemental Information

10.7717/peerj.18516/supp-1 Supplemental Information 1 Raw data.

10.7717/peerj.18516/supp-2 Supplemental Information 2 Data collection form/Questionnaire in English.

10.7717/peerj.18516/supp-3 Supplemental Information 3 Data collection form/Questionnaire in Arabic.

## Additional Information and Declarations

### Data Availability

The following information was supplied regarding data availability: The raw data is available in the Supplemental File.
