# Supplementary material for: Assessment of patient knowledge and perceptions towards orthodontic treatment in the Aljouf Region, Saudi Arabia: a cross-sectional study
Source: PeerJ. 2024 Nov 13;12:e18516. doi: 10.7717/peerj.18516 (PMC11568820; doi:10.7717/peerj.18516)
Supplement: Supplemental Information 2 [file peerj-12-18516-s002.pdf]

Data collection form

Part 1: Background characteristics

Age (Specify in years)

Gender

1. Male
2. Female

Education

1. No formal education
2. Up to school
3. University and above

Occupation

1. Government sector job
2. Private sector
3. Business/Self-employed
4. Student
5. Unemployed

Monthly family income:

1. Less than 5000 SAR
2. 5000 to 7000 SAR
3. More than 7000 SAR

Smoking:

1. Yes – Daily
2. Yes – Rarely
3. Never

Residence:

1. City/town
2. Rural/village

Have you received orthodontic treatment before?

1. Yes
2. No

Part 2: Questions related to knowledge: You may choose the best answer according to your knowledge:

1. What is the primary goal of orthodontic treatment?
   - A) To improve oral hygiene
   - B) To straighten teeth and correct bite issues
   - C) To prevent cavities
   - D) To treat gum disease

2. Which type of orthodontic appliance is typically worn at night to prevent teeth from shifting?
   - A) Braces
   - B) Retainer
   - C) Aligner
   - D) Headgear
3. What is a potential side effect of orthodontic treatment?
   - A) Increased risk of cavities
   - B) Decreased risk of gum disease
   - C) Improved speech

D) Reduced jaw pain

4. How often should you visit your orthodontist for adjustments during treatment?
   A) Once a year
   B) Once every six months
   C) Once a month
   D) Once every two years
5. At what age is orthodontic treatment typically initiated?
   A) Infancy
   B) Adolescence
   C) Young adulthood
   D) Elderly age
6. Which factor can influence the duration of orthodontic treatment?
   A) The patient's diet
   B) The patient's age
   C) The orthodontist's experience
   D) The number of orthodontic appliances used
7. What role do orthodontic retainers play after treatment?
   A) They prevent tooth decay
   B) They maintain the new position of the teeth
   C) They replace missing teeth
   D) They clean the teeth
8. What is one potential risk associated with orthodontic treatment?
   A) Reduced risk of tooth decay
   B) Improved self-esteem
   C) Allergic reactions to braces
   D) Enhanced facial symmetry
9. What can be a consequence of not wearing orthodontic retainers as instructed?
   A) Improved tooth alignment
   B) Tooth movement back to original positions
   C) Reduced risk of gum disease
   D) Enhanced smile aesthetics
10. How can orthodontic treatment impact overall oral health?
   A) It can improve chewing function
   B) It can worsen gum disease
   C) It can increase the risk of cavities
   D) It can lead to tooth loss

Part 3: Perception: You may choose from strongly disagree to strongly agree according to your perception.

Item	Strongly disagree	Disagree	Neutral	Agree	Strongly agree
Orthodontic treatment aims to improve both the appearance and function of the teeth and jaws					
Orthodontic treatment effectively enhances smile aesthetics.					

The benefits of orthodontic treatment justify the associated costs in terms of overall value and long-term outcomes.					
Orthodontic treatment is accessible and affordable for most individuals.					
The discomfort experienced during orthodontic treatment is manageable and worthwhile.					
Orthodontic treatment is an essential investment in long-term oral health.					
Orthodontic treatment significantly boosts self-confidence.					
Orthodontic treatment significantly reduces the risk of future dental problems.					
Orthodontic treatment enhances the overall quality of life					
A final informed decision to undergo orthodontics treatment or not, will be taken by me after discussing with the dentist.					
