# Supplementary material for: Assessment of patient knowledge and perceptions towards orthodontic treatment in the Aljouf Region, Saudi Arabia: a cross-sectional study
Source: PeerJ. 2024 Nov 13;12:e18516. doi: 10.7717/peerj.18516 (PMC11568820; doi:10.7717/peerj.18516)
Supplement: Supplemental Information 3 [file peerj-12-18516-s003.pdf]

الجزء الأول: الخصائص الشخصية

1. **العمر (حدد بالسنة):** ....

2. **الجنس:**

☐ ذكر ☐ أنثى

التعليم:

☐ لا يوجد تعليم رسمي. ☐ حتى المدرسة. ☐ الجامعة وما فوق.

3. **المهنة:**

☐ وظيفة في القطاع الحكومي. ☐ القطاع الخاص. ☐ تجارة/عمل حر. ☐ طالب. ☐ بدون عمل.

4. **الدخل الشهري للأسرة:**

☐ أقل من 5000 ريال سعودي ☐ من 5000 إلى 7000 ريال سعودي ☐ أكثر من 7000 ريال سعودي

5. **التدخين:**

☐ نعم – يومياً. ☐ نعم – نادراً. ☐ لا، أبداً.

6. **مكان الإقامة:**

☐ مدينة/بلدة. ☐ ريف/قرية.

7. **هل تلقيت علاج تقويم الأسنان من قبل؟**

☐ نعم. ☐ لا.

الجزء الثاني: المعرفة

تقييم مدى معرفتك تجاه علاج تقويم الأسنان يمكنك اختيار الإجابة الأفضل وفقًا لمعرفتك.

1. **ما هو الهدف الأساسي من علاج تقويم الأسنان؟**

أ) لتحسين النظافة الفموية

ب) لتقويم الأسنان وتصحيح مشكلات العضة

ج) للوقاية من التسوس

د) لعلاج أمراض اللثة

2. **أي نوع من أجهزة التقويم يُستخدم عادة في الليل لمنع الأسنان من التحرك؟**

أ) تقويم الأسنان

ب) الريتينر(الجهاز الثابت)

ج) الواقي الشفاف

د) الرأسية

3. **ما هو أحد الآثار الجانبية المحتملة لعلاج التقويم؟**

أ) زيادة خطر التسوس

ب) تقليل خطر أمراض اللثة

ج) تحسين النطق

د) تقليل ألم الفك

4. **كم مرة يجب أن تزور طبيب التقويم للتعديلات خلال العلاج؟**

أ) مرة واحدة في السنة

ب) مرة كل ستة أشهر

ج) مرة كل شهر

د) مرة كل سنتين

5. **في أي عمر يتم عادة بدء علاج التقويم؟**

أ) الرضاعة

ب) المراهقة

ج) الشباب

د) العمر المتقدم

6. **أي عامل يمكن أن يؤثر على مدة علاج التقويم؟**

أ) نظام المريض الغذائي

ب) عمر المريض

ج) خبرة الطبيب المعالج

د) عدد أجهزة التقويم المستخدمة

7. **ما الدور الذي يلعبه جهاز الاحتفاظ بعد العلاج؟**

أ) يمنع تسوس الأسنان

ب) يحافظ على الوضعية الجديدة للأسنان

ج) يحل محل الأسنان المفقودة

د) ينظف الأسنان

8. **ما هو أحد المخاطر المحتملة المرتبطة بعلاج التقويم؟**

أ) تقليل خطر التسوس

ب) تحسين الثقة بالنفس

ج) ردود فعل تحسسية للتقويم

د) تحسين التناظر الوجهي

9. **ماذا يمكن أن يكون عواقب عدم ارتداء جهاز الاحتفاظ كما يُوصبه؟**

أ) تحسين محاذاة الأسنان

ب) عودة الأسنان إلى وضعيتها الأصلية

ج) تقليل خطر الإصابة بأمراض اللثة

د) تحسين جمالية الابتسامة

10. **كيف يمكن لعلاج التقويم أن يؤثر على الصحة الفموية العامة؟**

أ) يمكن أن يحسن وظيفة المضغ

ب) يمكن أن يؤدي إلى تفاقم أمراض اللثة

ج) يمكن أن يزيد من خطر التسوس

د) يمكن أن يؤدي إلى فقدان الأسنان

الجزء الثالث: الإدراك

تقييم مدى إدراكك تجاه علاج تقويم الأسنان، يمكنك الاختيار من "أوافق بشدة" إلى "أعارض بشدة" وفقًا لإدراكك.

	أعارض بشدة	أعارض	محايد	أوافق	أوافق بشدة
1. العلاج التقويمي يهدف إلى تحسين مظهر ووظيفة الأسنان والفكين.					
2. العلاج التقويمي يعزز بشكل فعّال جماليات الابتسامة.					
3. الفوائد المترتبة على العلاج التقويمي تبرر التكاليف المرتبطة به من حيث القيمة الإجمالية والنتائج طويلة الأمد.					
4. العلاج التقويمي يعتبر متاحًا وبأسعار معقولة لمعظم الأشخاص.					
5. الإزعاج الذي يُختبر خلال العلاج التقويمي يمكن التحكم فيه ويستحق التجربة.					
6. العلاج التقويمي استثمار ضروري في صحة الفم على المدى الطويل.					
7. العلاج التقويمي يعزز بشكل كبير الثقة بالنفس.					
8. العلاج التقويمي يقلل بشكل ملحوظ من خطر المشاكل السنية في المستقبل.					
9. العلاج التقويمي يحسن بشكل عام جودة الحياة.					
10. القرار النهائي والمستنير بشأن الخضوع للعلاج التقويمي من عدمه، سيتخذ من قبلي بعد مناقشته مع طبيب الأسنان.					
